# Logistic regression analysis of risk factors for Respiratory Distress Syndrome in Late Preterm Infants

**DOI:** 10.12669/pjms.41.2.9796

**Published:** 2025-02

**Authors:** Hongbin Zhu, Yueyi Wang, Xuexu Wei, Wei Shi, Haiwei Yin, Caiyun Gao

**Affiliations:** 1 Hongbin Zhu, Department of Paediatrics, Maternity & Child Care Center of Qinhuangdao, Qinhuangdao 066000, Hebei, China; 2 Yueyi Wang, Department of Paediatrics, Maternity & Child Care Center of Qinhuangdao, Qinhuangdao 066000, Hebei, China; 3 Xuexu Wei, Department of Paediatrics, Maternity & Child Care Center of Qinhuangdao, Qinhuangdao 066000, Hebei, China; 4 Wei Shi, Department of Paediatrics, Maternity & Child Care Center of Qinhuangdao, Qinhuangdao 066000, Hebei, China; 5 Haiwei Yin, Department of Paediatrics, Maternity & Child Care Center of Qinhuangdao, Qinhuangdao 066000, Hebei, China; 6 Caiyun Gao, Department of Paediatrics, Maternity & Child Care Center of Qinhuangdao, Qinhuangdao 066000, Hebei, China

**Keywords:** Premature infants, Respiratory distress syndrome, Risk factors, Diabetes, Cesarean section

## Abstract

**Objective:**

To find out the risk factors for respiratory distress syndrome in late preterm infants.

**Methods:**

This was a retrospective study. A total of 1605 premature infants born in Maternity & Child Care Center of Qinhuangdao from January 2020 to June 2023 were selected as the research subjects. They were divided into RDS group and non RDS group based on the presence or absence of respiratory distress syndrome(RDS). The clinical pathological characteristics of the two groups of patients were compared. Logistic regression analysis was used to analyze the risk factors for respiratory distress syndrome in late stage premature infants.

**Results:**

The results of univariate analysis showed that there was no statistically significant difference in neonatal weight, placental abnormalities, gestational hypertension, and maternal age between the RDS group and the non RDS group(P>0.05). There were significant differences in gender, whether premature rupture of membranes, whether cesarean section, and diabetes during pregnancy(P<0.05). The binary logistic regression analysis showed that gender(male), cesarean section(yes) and diabetes in pregnancy(yes) were all risk factors for RDS in preterm infants (P<0.05). Premature rupture of membranes is a protective factor for RDS in premature infants (P<0.05). Gender (male), cesarean section(yes), and diabetes in pregnancy(yes) were all risk factors for poor prognosis of RDS children(P<0.05). Premature rupture of membranes (PROM) is a protective factor for poor prognosis in premature infants (P<0.05).

**Conclusion:**

Male sex, cesarean section, and diabetes in pregnancy may be the risk factors for RDS and poor prognosis of premature infants.

## INTRODUCTION

Respiratory distress syndrome (RDS) is a common pediatric disease, and late preterm infants are prone to RDS due to incomplete lung development.^1^ To investigate the risk factors of RDS in late preterm infants, logistic regression analysis was used in this study to screen and analyze the relevant factors. In clinical practice, late preterm infants are often subject to multiple risk factors, such as gestational complications, asphyxia at birth, and postpartum infection, which may act alone or in combination to contribute to the development of RDS.^2^-^5^ Logistic regression analysis can quantitatively assess the impact of these factors on the occurrence of RDS, and calculate the corresponding odds ratio and 95% confidence interval of each factor, so as to better understand the characteristics of risk factors. Therefore, this study was designed to investigate the risk factors of RDS in late preterm infants by selecting a total of 1,605 premature infants born in Maternity & Child Care Center of Qinhuangdao from January 2020 to January 2023 as the research subjects.

## METHODS

This was a retrospective study. A total of 1605 premature infants born in Maternity & Child Care Center of Qinhuangdao from January 2020 to June 2023 were selected as the research subjects and divided into the RDS group(n=190) and non-RDS group(n=1415) according to the presence or absence of respiratory distress syndrome (RDS). Data were retrieved from our hospital information and management system and collected their various information.

### Ethical Approval:

The study was approved by the Institutional Ethics Committee of Maternity & Child Care Center of Qinhuangdao (No.:QHDFY-2023031009; Date: March 31, 2023), and written informed consent was obtained from the guardians of the participants.

### Inclusion criteria:

- Delivery at our hospital.
- Admitted to neonatal intensive care unit within six hours of birth.
- Family members signed informed consent.

### Exclusion criteria:

- Patients without complete clinical data.
- Patients who withdrew from the study for some reason.

### Analysis method:

The general data, neonatal weight, placental abnormalities, gestational hypertension, maternal age, gender, whether premature rupture of membranes, whether cesarean section, gestational diabetes, and other related factors of the RDS group and the non-RDS group were analyzed by referring to the relevant literature, so as to explore the high-risk factors and protective factors of RDS in premature infants. One hundred and ninety premature infants with RDS were divided into the cured/improved group (n=152) and the abandoned/dead group (n=38) according to their prognosis. The general data, neonatal weight, abnormal placenta, gestational hypertension, maternal age, gender, premature rupture of membranes (PROM), cesarean section, gestational diabetes, and other related factors were analyzed to explore the high-risk factors and protective factors of the prognosis of RDS in premature infants.

### Statistical Analysis:

SPSS 22.0 was used for statistical analysis. Measurement data following normal distribution were expressed as (*χ̅*±*S*), and the independent sample t-test was performed; categorical variables were expressed as the number of cases and percentage, and χ^2^ test was conducted; P< 0.05 was considered statistically significant.

## RESULTS

Univariate analysis showed no significant difference in neonatal weight, placental abnormalities, gestational hypertension, and maternal age between the RDS and non-RDS groups (P > 0.05). However, there were significant differences in gender, premature rupture of membranes (PROM), cesarean section, and gestational diabetes (P < 0.05) (Table-I). Binary logistic regression analysis with “RDS” as the dependent variable (assigned value: 0 = yes, 1 = no) and “gender, premature rupture of membranes (PROM), cesarean section, and gestational diabetes” as independent variables showed that gender (male), cesarean section (yes), and gestational diabetes (yes) were all risk factors for RDS in premature infants (P < 0.05). Meanwhile, PROM (yes) was a protective factor for RDS in premature infants (P < 0.05) (Table-II).

**Table-I:** Univariate Analysis of RDS in Premature Infants.

Factor	n	RDS Group(190)	Non-RDS Group(1415)	χ^2^	P
***Gender***				20.870	<0.001
Male	901	136(15.09)	765(84.91)		
Female	704	54(7.67)	650(92.33)		
***Neonatal Weight***				0.002	0.967
<2500g	834	99(11.87)	735(88.13)		
≥2500g	771	91(11.80)	680(88.20)		
***Placental Abnormalities***				3.154	0.076
Yes	154	25(16.23)	129(83.77)		
No	1451	165(11.37)	1286(88.63)		
***PROM***				25.823	<0.001
Yes	644	44(6.83)	600(93.17)		
No	961	146(15.19)	815(84.81)		
***Cesarean Section***				49.818	<0.001
Yes	1083	171(15.79)	912(84.21)		
No	522	19(3.64)	503(96.36)		
***Gestational Hypertension***				0.308	0.579
Yes	421	53(12.59)	368(87.41)		
No	1184	137(11.57)	1047(88.43)		
***Gestational Diabetes***				4.134	0.042
Yes	489	70(14.31)	419(85.69)		
No	1116	120(10.75)	996(89.25)		
***Maternal Age***				0.007	0.935
≥35	350	41(11.71)	309(88.29)		
<35	1255	149(11.87)	1106(88.13)		

**Table-II:** Multivariate Logistic Regression Analysis.

Variable	B	S.E.	Wald	df	P	OR	95% CI	

							Minimum	Maximum
Gender	0.723	0.173	17.424	1	<0.001	2.061	1.468	2.894
PROM (yes)	-0.869	0.183	22.404	1	<0.001	0.420	0.293	0.601
Cesarean Section (yes)	1.560	0.250	38.923	1	<0.001	4.758	2.915	7.766
Gestational Diabetes (yes)	0.327	0.161	4.108	1	0.043	1.387	1.011	1.902
Constant	0.201	0.549	0.135	1	0.714	1.223		

Univariate analysis suggested no significant difference in neonatal weight, placental abnormalities, gestational hypertension, and maternal age between the RDS and non-RDS groups (P > 0.05). However, significant differences were observed in gender, premature rupture of membranes (PROM), cesarean section, and gestational diabetes (P < 0.05) (Table-III). The binary logistic regression analysis was performed with the “Abandoned/Dead Group” as the dependent variable (assigned value: 0 = yes, 1 = no) and “gender, premature rupture of membranes (PROM), cesarean section, and gestational diabetes” as independent variables, which showed that gender (male), cesarean section (yes), and gestational diabetes (yes) were all risk factors for poor prognosis in infants with RDS (P < 0.05). Meanwhile, PROM (yes) was a protective factor for poor prognosis in premature infants (P < 0.05) (Table-IV).

**Table-III:** Univariate Analysis of Prognosis of RDS in Premature Infants.

Factor	n	Cured/Improved Group(152)	Abandoned/Dead Group(38)	χ^2^	P
***Gender***				6.216	0.013
Male	136	115(84.56)	21(15.44)		
Female	54	37(68.52)	17(31.48)		
***Neonatal Weight***				0.005	0.942
<2500g	99	79(79.8)	20(20.2)		
≥2500g	91	73(80.22)	18(19.78)		
***Placental Abnormalities***				0.000	1.000
Yes	25	20(80)	5(20)		
No	165	132(80)	33(20)		
***PROM***				6.218	0.013
Yes	44	41(93.18)	3(6.82)		
No	146	111(76.03)	35(23.97)		
***Cesarean Section***				18.947	<0.001
Yes	171	144(84.21)	27(15.79)		
No	19	8(42.11)	11(57.89)		
***Gestational Hypertension***				0.026	0.871
Yes	53	42(79.25)	11(20.75)		
No	137	110(80.29)	27(19.71)		
***Gestational Diabetes***				5.089	0.024
Yes	70	50(71.43)	20(28.57)		
No	120	102(85)	18(15)		
***Maternal Age***				0.008	0.930
≥35	41	33(80.49)	8(19.51)		
<35	149	119(79.87)	30(20.13)		

**Table-IV:** Multivariate Logistic Regression Analysis.

Variable	B	S.E.	Wald	df	P	OR	95% CI	

							Minimum	Maximum
Gender(M)	1.050	0.424	6.128	1	0.013	2.857	1.244	6.56
PROM (Yes)	1.691	0.665	6.468	1	0.011	5.424	1.474	19.962
Cesarean Section (Yes)	2.031	0.566	12.858	1	<0.001	7.622	2.512	23.132
Gestational Diabetes (Yes)	-1.019	0.413	6.083	1	0.014	0.361	0.161	0.811
Constant	-6.610	1.666	15.741	1	<0.001	0.001		

## DISCUSSION

In this study, 1,605 premature infants born in Maternity & Child Care Center of Qinhuangdao from January 2020 to January 2023 were selected as the research subjects and divided into the RDS and non-RDS groups according to the presence or absence of respiratory distress syndrome (RDS). Univariate analysis showed no significant difference in neonatal weight, placental abnormalities, gestational hypertension, and maternal age between the RDS and non-RDS groups (P > 0.05). However, significant differences were observed in gender, premature rupture of membranes (PROM), cesarean section, and gestational diabetes (P< 0.05).

Meanwhile, the binary logistic regression analysis indicated that gender (male), cesarean section (yes), and gestational diabetes (yes) were all risk factors for RDS in preterm infants (P < 0.05), while PROM (yes) was a protective factor (P < 0.05). The analysis suggested that men are more prone to NRDS than women, partly due to the differences in hormonal regulation. In women, estrogen can promote lung maturation, while androgen has no significant effect on lung maturation.^6^ Therefore, male preterm infants may be more prone to incomplete lung development due to a lack of estrogen protection, resulting in the development of NRDS. Also known as neonatal respiratory distress syndrome or neonatal hyaline membrane disease, respiratory distress syndrome in premature infants is a serious lung disease caused by pulmonary surfactant deficiency, which leads to extensive alveolar collapse and damage in both lungs, ultimately resulting in acute respiratory failure.^7^-^9^

This disease is commonly seen in premature infants, especially those with a small gestational age (37 weeks old < 5% and 15% for 32-34 weeks old). Neonatal respiratory distress syndrome may significantly affect the life and health of newborns, and it is prone to cause complications such as patent ductus arteriosus, persistent pulmonary hypertension, and pulmonary hemorrhage due to its high severity.^10^ Additionally, bronchopulmonary dysplasia may occur in the long term, and severe cases may lead to multiple organ failure and even death without timely and effective treatment.^11^ In contrast, late preterm respiratory distress syndrome is a pediatric disorder caused by incomplete lung development.

Meanwhile, late preterm infants are prone to dyspnea, respiratory distress, and other problems due to immature lung development. In clinical practice, respiratory distress syndrome is mainly characterized by symptoms such as increased respiration, difficulty in breathing, and respiratory groaning, and some infants may also develop symptoms such as inspiratory depression of the suprasternal and inferior fossae and cyanosis, which may lead to respiratory failure, hypoxemia, and acidosis in severe cases.^12^-^14^ Additionally, cesarean section may exhibit some effects on the fetus. During spontaneous delivery, the lungs of the fetus are squeezed as it passes through the birth canal, which helps the fetus expel fluid from the lungs and promotes lung maturation. Cesarean section, however, skips this process and may ultimately lead to incomplete fetal lung development and increase the risk of NRDS.^15^-^17^ Diabetic pregnant women have high blood glucose, which leads to increased blood glucose in the fetus, in which case the fetus grows obese and huge but the lungs do not necessarily mature.

Moreover, insulin antagonizes the effects of adrenocorticotropic hormone and affects lung development. Both insulin resistance and inflammatory responses may contribute to the decreased ability of alveolar cells to synthesize PS, thereby increasing the risk of NRDS.^18^-^20^ Causes of PROM include infection, polyhydramnios, and incompetence of the internal orifice of the uterus. If PROM occurs, amniotic fluid may pass from the uterus into the mother’s body, resulting in sudden exposure of the fetus to an unprotected environment, thus increasing the risk of developing NRDS. However, PROM does not always lead to the development of NRDS. Some studies have shown that if PROM occurs after 34 weeks of gestation, the risk of developing NRDS is reduced, possibly because PROM makes it possible for the fetus to be born in a more mature state, thus reducing the risk of developing NRDS.

### Limitations:

It includes no long-term follow-up was conducted. In future studies follow-up time should be increased to validate the findings of this study.

## CONCLUSION

Male gender, cesarean section, and gestational diabetes may be risk factors for RDS in premature infants, and attention should be paid to such factors in clinical practice along with further analysis, thereby providing more help for pregnant women subjecting to these conditions.

### Authors’ Contributions:

**HZ** and **XW: Concept, study design,** Carried out the studies, data collection, and drafted the manuscript, and are responsible and accountable for the accuracy or integrity of the work.

**YW** and **WS:** Literature search, performed the statistical analysis and participated in its design.

**HY** and **CG:** Participated in acquisition, analysis, or interpretation of data and draft the manuscript. All authors read and approved the final manuscript.
